# Modelling the Transmission Dynamics and Control of Mumps in Mainland China

**DOI:** 10.3390/ijerph15010033

**Published:** 2017-12-26

**Authors:** Yong Li, Xianning Liu, Lianwen Wang

**Affiliations:** 1 Key Laboratory of Eco-Environments in Three Gorges Reservoir Region (Ministry of Education), School of Mathematics and Statistics, Southwest University, Chongqing 400715, China; yongli@yangtzeu.edu.cn; 2 School of Information and Mathematics, Yangtze University, Jingzhou 434023, China; 3 Department of Mathematics, Hubei University for Nationalities, Enshi 445000, China; wanglianwen1987@hotmail.com

**Keywords:** basic reproductive number, global stability, sensitivity analysis, SVEILR mumps model, vertical transmission

## Abstract

Mumps is a common childhood viral disease and children have been vaccinated throughout the world since 1967. The incidence of mumps has increased with more than 300,000 young people infected with mumps annually in mainland China since 2005. Therefore, we designed and analyzed long-term mumps surveillance data in an SVEILR (susceptible–vaccinated–exposed–severely infectious–mildly infectious–recovered) dynamic transmission model with optimized parameter values to describe the dynamics of mumps infections in China. There were 18.02% of mumps infected young adults seeking medical advice. The vaccine coverage has been insufficient in China. Young adults with frequent contact and mild infection were identified as a major driver of mumps epidemics. The reproduction number of mumps was determined 4.28 in China. Sensitivity analysis of the basic reproduction number and the endemic equilibrium was conducted to evaluate the effectiveness of mumps control measures. We propose to increase the vaccine coverage and make two doses of MMR (Measles, mumps and rubella) vaccines freely available in China.

## 1. Introduction

Mumps is best known as a common childhood viral disease and is highly contagious to human beings. Initial signs and symptoms often include fever, muscle pain and headache, then usually followed by painful swelling of one or both parotid salivary glands [1]. The disease caused by the mumps virus, the causative agent of mumps infection, is an enveloped RNA virus [2]. Since the disease is generally benign and self-resolving, its mortality is rare, but aseptic meningitis can affect 10% of case-patients [3]. Mumps is a significant cause of pediatric deafness, and up to 37% of post-pubertal males develop orchitis, 13% of whom have impaired fertility [1].

Due to the lack of vaccines, most teenagers have been mainly infected by those patients aged 4–15 years. Transmission of the virus is by direct physical contact, droplet spread, or contaminated fomites [4,5,6]. The incubation period is about 15 to 24 days (median is 19 days) [7]. Infected patients become the most contagious in 1–2 days before onset of clinical symptoms and continue so for a few days afterwards. Generally speaking, the infectious period is about eight days [8], and the patients will recover between 10 to 14 days [9,10].

Mumps has periodic outbreaks and no specific antiviral therapy, and treatment is just mostly symptomatic and supportive [11]. In 1967, the attenuated mumps virus vaccine was licensed in the United States [12]. In addition, mumps is preventable by two doses of the mumps vaccine in many developed countries nowadays [13]. They includes it in their immunization programs, often in combination with measles and the rubella vaccine [14,15]. Nowadays, children aged 18–24 months just routinely receive one dose of the measles–mumps–rubella (MMR) vaccine free of charge in China, and National Health and Family Planning Commission of the People’s Republic of China (NHFPC) doesn’t emphasize inoculating the second dose of MMR. In recent years, there are more than 300,000 young people infected with mumps every year in China [16,17].

The number of mumps patients is just smaller than hand, foot and mouth disease in all pediatric infectious diseases [16,17]. Compared with other usual vaccine-preventable diseases, such as measles and pertussis, mumps is more common. Due to its less severe acute manifestation, mumps have been somewhat neglected. Nevertheless, the UK and USA have been inspired a new interest in mumps by some large outbreaks. In the UK, a large mumps epidemic began in 2004 and reached the peak in 2005 with about 56,000 reported cases [18,19]. Most of these cases were in young adults attending colleges or universities [20]. In 2006, the USA underwent a multi-state outbreak involving 6584 reported cases, with the highest attack rate among persons 18–24 years old, many of whom were college students [12]. In affected colleges, most case-patients had been inoculated with a second dose of the MMR vaccine more than 10 years previously [21,22]. This was the first large-scale US mumps outbreak among the population with two-dose vaccines. From then on, people realized that even the two doses of the vaccine could not completely control mumps either.

Mathematical models have become vital tools in understanding the spread and control of infectious diseases well. By setting up a suitable epidemic model, we can put forward a lot of practical prevention and control measures to curb the epidemic of disease. So far, there have been a few papers using dynamic models to study mumps [23,24]. Qu [23] proposed a non-autonomous SVEILHR (susceptible–vaccinated–exposed–mild infectious–severe infectious–hospitalized–recovered) model with a seasonal varying transmission rate to describe the epidemic of mumps, and suggested improving vaccine coverage and providing two doses of MMR (Measles, mumps and rubella) vaccine by the government in China. Liu [24] studied the effects of heterogeneity on mumps spread by constructing a multi-group SVEIAR (susceptible–vaccinated–exposed –symptomatic–asymptomatic–recovered) mumps model with asymptomatic infection, general vaccinated and exposed distributions and established the threshold dynamics of the model.

The basic structures of this paper are as follows. In the next section, an SVEILR mumps model is formulated, and we give the basic reproduction number $R_{0}$ and the existence of equilibria. Section 3 discusses the global stability of the model. In Section 4, we advance the optimal parameters, simulation of the real data from 2009 to 2014, and the prediction results of 2014 and 2015. Sensitivity analysis of the basic reproduction number $R_{0}$ and endemic equilibrium $P^{*}$ are carried out in Section 5. We conclude with some discussions in Section 6 about the role of vaccine and the preventive measures on mumps. We close with a conclusions section.

## 2. The Mumps Model and Basic Reproduction Number $R_{0}$

**Figure 1:**
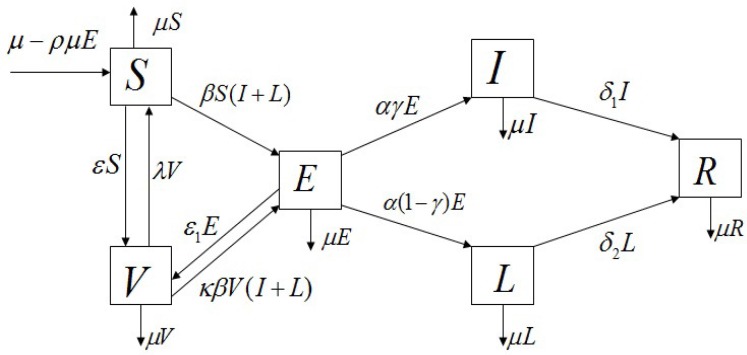
Flowchart of mumps transmission in a population.

We propose a mathematical model to understand the transmission dynamics and prevalence of mumps in mainland China. Since we will simulate real data for half a year, we think the total population is constant in a short period of time. We assume that the birth rate equals the natural death rate, denoted by μ. The model is constructed based on the characteristics of mumps transmission; therefore, the population associated with mumps is divided into six epidemiological sub-classes: the proportion susceptible to total population (*S*), the proportion of vaccinated to total population (*V*), the proportion exposed to total population (*E*, infected but not infectious), the proportion severely infectious to total population (*I*, severely infectious requiring medical attention), the proportion mildly infectious to total population (*L*, mild infections, including both asymptomatic and those with mild symptoms and self-care), and the proportion recovered to total population (*R*), subject to the restriction S+V+E+I+L+R=1. The transmission dynamics associated with these sub-classes are illustrated in Figure 1.

Considering the vertical transmission, we assume that a fraction ρ of the offspring from the exposed parents is born into the exposed class *E*. Consequently, the birth flux into the exposed class is given by ρμE and the birth flux into the susceptible class is given by μ−ρμE. The other defined parameters in the model Equation (2) are listed below:

- β: transmission rate,
- λ: loss of vaccination rate,
- ε: vaccine coverage of the susceptible,
- $\varepsilon_{1}$: vaccine coverage of the exposed,
- κ: invalid vaccination rate,
- α: rate moving from exposed to severe or mild infectious,
- γ: proportion of the severe infections seeking medical advice,
- $\delta_{1}$: rate moving from severe infectious to recovered,
- $\delta_{2}$: rate moving from mild infectious to recovered.

To simplify the research, we don’t consider the spatial stratified heterogeneity of the population and relevant determinants, and consider that the mumps model has homogeneous mixing, and an individual has an equal chance of contacting any individual among the population. The SVEILR mumps model is given by six ordinary differential equations:

(1) $$\begin{array}{c} \left\{\begin{array}{c} \frac{dS}{dt}=\mu -\rho \mu E-\beta S\left(I+L\right)+\lambda V-\left(\varepsilon +\mu \right)S,\\ \frac{dV}{dt}=\varepsilon S+\varepsilon_{1}E-\lambda V-\kappa \beta V\left(I+L\right)-\mu V,\\ \frac{dE}{dt}=\beta S\left(I+L\right)+\rho \mu E+\kappa \beta V\left(I+L\right)-\left(\alpha +\varepsilon_{1}+\mu \right)E,\\ \frac{dI}{dt}=\alpha \gamma E-\left(\delta_{1}+\mu \right)I,\\ \frac{dL}{dt}=\alpha \left(1-\gamma \right)E-\left(\delta_{2}+\mu \right)L,\\ \frac{dR}{dt}=\delta_{1}I+\delta_{2}L-\mu R. \end{array}\right. \end{array}$$

A person was infected with the virus and then fully recovered. After that, he is typically immune for life [20]. The first five equations are independent of *R* in Equation (1), and then it suffices to study the following sub-system:

(2) $$\begin{array}{c} \left\{\begin{array}{c} \frac{dS}{dt}=\mu -\rho \mu E-\beta S\left(I+L\right)+\lambda V-\left(\varepsilon +\mu \right)S,\\ \frac{dV}{dt}=\varepsilon S+\varepsilon_{1}E-\lambda V-\kappa \beta V\left(I+L\right)-\mu V,\\ \frac{dE}{dt}=\beta S\left(I+L\right)+\rho \mu E+\kappa \beta V\left(I+L\right)-\left(\alpha +\varepsilon_{1}+\mu \right)E,\\ \frac{dI}{dt}=\alpha \gamma E-\left(\delta_{1}+\mu \right)I,\\ \frac{dL}{dt}=\alpha \left(1-\gamma \right)E-\left(\delta_{2}+\mu \right)L. \end{array}\right. \end{array}$$

The biologically feasible region of Equation (2) is $\Omega =\{\left(S,V,E,I,L\right)\in R_{+}^{5}:0\leq S+V+E+I+L\leq 1\}$, which can be verified as positively invariant (i.e., given non-negative initial values in Ω, all solutions to Equation (2) have non-negative components and stay in Ω for t≥0) and globally attracting in $R_{+}^{5}$ with respect to Equation (2). Therefore, we restrict our attention to the dynamics of Equation (2) in Ω.

It is easy to see that model Equation (2) always has a disease-free equilibrium $P_{0}=\left(S_{0},V_{0},0,0,0\right)$, where

(3) $$\begin{array}{c} S_{0}=\frac{\lambda +\mu }{\varepsilon +\lambda +\mu },V_{0}=\frac{\varepsilon }{\varepsilon +\lambda +\mu }. \end{array}$$

According to the next generation matrix developed by van den Driessche and Watmough [25], we define the basic reproduction number of model Equation (2) as

(4) $$\begin{array}{c} R_{0}=\frac{\rho \mu }{\varepsilon_{1}+\alpha +\mu }+\frac{\beta \alpha (\lambda +\mu +\kappa \varepsilon )}{\left(\varepsilon_{1}+\alpha +\mu \right)\left(\varepsilon +\lambda +\mu \right)}\left(\frac{\gamma }{\delta_{1}+\mu }+\frac{1-\gamma }{\delta_{2}+\mu }\right)\;\\ =R_{E}+R_{I}+R_{L}, \end{array}$$

where $R_{E}=\frac{\rho \mu }{\varepsilon_{1}\;+\;\alpha \;+\;\mu }$, $R_{I}=\frac{\beta \alpha \gamma (\lambda \;+\;\mu \;+\;\kappa \varepsilon )}{\left(\varepsilon_{1}\;+\;\alpha \;+\;\mu \right)\left(\varepsilon \;+\;\lambda \;+\;\mu \right)\left(\delta_{1}\;+\;\mu \right)}$, $R_{L}=\frac{\beta \alpha (1-\gamma )(\lambda \;+\;\mu \;+\;\kappa \varepsilon )}{\left(\varepsilon_{1}\;+\;\alpha \;+\;\mu \right)\left(\varepsilon \;+\;\lambda \;+\;\mu \right)\left(\delta_{2}\;+\;\mu \right)}$, which represent the average numbers of the infected individuals by a single exposed parents, severely infectious, and mildly infectious individuals in a fully susceptible population, respectively.

The basic reproduction number $R_{0}$ represents for the average number of new infections brought out by one infectious during the initial patient’s infectious (not sick) period [26]. If $R_{0}>1$, model Equation (2) has a unique endemic equilibrium $P^{*}=\left(S^{*},V^{*},E^{*},I^{*},L^{*}\right)$. Here, we do not give the exact expression of $P^{*}$, the detailed analysis can be seen in the Appendix A. Then, we have the following proposition:

**Proposition** **1.** Model Equation (2) always has a disease-free equilibrium $P_{0}$; and, if $R_{0}>1$, model Equation (2) has a unique endemic equilibrium $P^{*}$.

## 3. Mathematical Analysis Results

Regarding the stability of the disease-free equilibrium $P_{0}$ and endemic equilibrium $P^{*}$, we have the following Theorems. The detailed proof process can be seen in the Appendix B.

**Theorem** **1.** If $R_{0}<1$, then $P_{0}$ is stable, and, if $R_{0}>1$, then $P_{0}$ is unstable.

**Theorem** **2.** The disease-free equilibrium $P_{0}$ of model Equation (2) is globally asymptotically stable if $R_{0}\leq 1$.

The above results show that mumps will be eliminated from the community if the epidemiological threshold $R_{0}$ can be brought to a value less than unity.

**Theorem** **3.** If $R_{0}>1$, for $\rho =\varepsilon_{1}=0$, model Equation (2) has a unique endemic equilibrium $P^{*}=\left(S^{*},V^{*},E^{*},I^{*},L^{*}\right)$, which is globally asymptotically stable.

When $\rho \neq 0,\varepsilon_{1}\neq 0$, we do not testify the global stability of the model Equation (2). Nevertheless, using parameter estimation in the next section, the values of ρ and $\varepsilon_{1}$ are very small, almost negligible. It shows that Theorem 3 still have great significance.

Mathematical analysis shows that the basic reproduction number $R_{0}$ is a sharp threshold completely determining the global dynamics of model Equation (2): if $R_{0}\leq 1$, the disease-free equilibrium $P_{0}$ is globally asymptotically stable and the mumps dies out; if $R_{0}>1$, the endemic equilibrium $P^{*}$ is globally asymptotically stable, namely, the mumps persists at the endemic equilibrium state if only it initially exists.

## 4. Data and Parameter Estimation

### 4.1. Data Analysis

**Figure 2:**
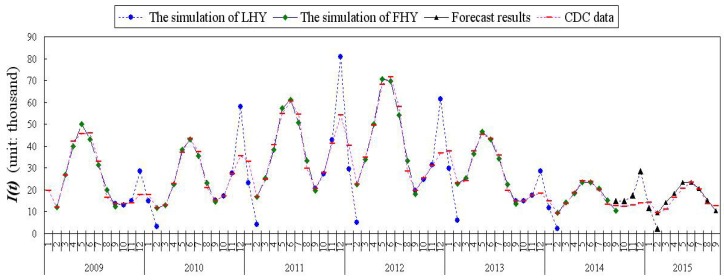
The comparison chart of the data of mumps in China and simulation results by model Equation (2).

It was reported by the Ministry of Health of the People’s Republic of China that mumps included made the list as a Category C Infectious Disease (Monitoring and Managing of Infectious Disease) in 1989 [27]. On the basis of the Chinese Center for Disease Control and Prevention (China’s CDC), using month as the period does statistical work on the patients who were infected by mumps (see Figure 2) [16,17]. China’s CDC publishes the surveillance data of mumps from each province in mainland China except Hong Kong, Macao and Taiwan. From the network direct reporting surveillance data, we can acquire a lot of detailed information from cases, such as the area code, gender, occupation, date of birth, date of onset, date of diagnosis, address, and, especially, classification of disease, which is labeled as clinically diagnosed cases.

According to the data of mumps analysis, it is not difficult to find that the epidemic of mumps has certain regularity. Judging by larger number of mumps cases, we can find a peak between February and September, another peak appears between September of this year to February of the next year, and the trough appears in February and September each year [28]. In fact, it is associated with the holiday scheme of the elementary and secondary schools in China. Every year in February, which is just after the lunar New Year, all primary and secondary schools will end more than a month of winter vacation. Similarly, every year in July to September, all schools have a summer vacation to rest for two months. This time happens to be when the mumps go through the trough. On account of the patients with mumps being mainly 4 to 15 years old, we can conclude that the main cause of the mumps epidemic in China is close contact among the students in their schools. Considering Chinese summer vacation and winter vacation, in order to facilitate the fitting, we therefore divide the reported data of each year into two parts: one is the first half of the year (from February to September), denoted by FHY (the first half of the year), and the other is the latter half of the year (from September of this year to February of the next year), denoted by LHY (the latter half of the year). Therefore, the data of the February and September are simulated twice.

### 4.2. Parameter Estimation

Next, the model parameters are estimated as follows:

(1) Assuming that the person’s natural death follows a uniform distribution, natural death rate is then calculated as $\mu =1/\left(74.83\times 365\right)=3.6613\times 10^{-5}$ [17,29].
(2) Note that κ and ρ are relatively small and almost equal to 0 by simulation, and we choose κ=ρ=0. It shows that invalid vaccination and vertical transmission are not main factors for the mumps epidemic. The course of treatment for the infectious is about 12 days (range, 10–14) [9,10,16]. Then, setting $\delta_{1}=1/12$, we also assume $\delta_{2}=1/12$.
(3) By applying a chi-square test to fit annual data (six data points in FHY, eight data points in LHY), at most six parameters (or initial values) every time can be fit into the first half of the year, and at most four parameters (or initial values) every time can be fit into the latter half of the year. We thus consider $\varepsilon =\varepsilon_{1}$, E(0)=I(0)=R(0)=0.
(4) The remaining parameters and initial values (β, λ, ε, γ, S(0) and V(0)) in model Equation (2) are estimated through calculating the minimum sum of chi-square value (MSCV) [30].
(5) Assume that the population about mumps is 140,000 every month. Before fitting, one put the real data reducing 140,000 times in the simulation as the proportion severely infectious seeking medical advice to total population. In addition, after fitting, we can multiply by 140,000 to the estimation valves.

The data presented in Figure 2 refers to the clinical data from China’s CDC, denoted by *I*, and we can see the optimal parameter values and initial values of FHY and LHY from 2009 to 2014 in Table A1 and Table A2. In addition, the chi-square test of goodness of fit is shown in Appendix C. Here, we also calculate the basic reproduction number, $R_{0}$, the median and the arithmetic mean of the optimal parameters, which are estimated by MSCV as shown in Table 1. The difference between the same parameters is very small in FHY and LHY of different years. In addition, $R_{0}$ of each year is stable around 4, the range of $R_{0}$ is 2.6529–5.5563, the median of $R_{0}$ is 4.7497 and arithmetic mean of $R_{0}$ is 4.2806. This suggests that the endemic equilibrium of model Equation (2) is globally asymptotically stable. It is said that, in this current situation, mumps will continue to spread.

As we know, the vaccination rate of the susceptible is ε=ηφ, and the vaccination rate of the exposed is $\varepsilon_{1}\;=\;\eta_{1}\varphi_{1}$. Here, η and $\eta_{1}$ are defined to be the percentage of the number of vaccinations, and φ are $\varphi_{1}$ to defined to be the rate of progression to the vaccinated. Unfortunately, we can’t get the respective parameter values of η, $\eta_{1}$, φ and $\varphi_{1}$. We just obtain their products ε and $\varepsilon_{1}$ in model Equation (2) by MSCV.

Using model Equation (2) and the parameters from Table A1 and Table A2, one carries on the data fitting to the number of severely infectious seeking medical advice individuals, as shown in Figure 2, and the numerical results are found to be a good match with the data of mumps from 2009 to 2014. From 1960 to 1980, $R_{0}$ in the Netherlands, England and Wales was 11–14, and in various states of USA, $R_{0}$ was 4–7 [9]. In recent years, the literature estimates the basic reproduction number of mumps was about 6.5428 in China [23]. Edmunds [31] used the default matrix configuration to estimate $R_{0}$ for mumps was about 3.6–4.5 in many European countries (England and Wales, the Netherlands, Finland, Denmark, East Germany and Italy) from 1970–1990. Kanaan [32] used the matrix models with individual heterogeneity to estimate $R_{0}$ for mumps was 19.3 (95% credible region (CR) 4.0–31.5) in UK in 1986, if heterogeneity is not considered, $R_{0}$ was much smaller (about 4.44). In addition, by our model, we estimate that the arithmetic mean of $R_{0}$ of mumps is about 4.2806. In the current literature, $R_{0}$ for mumps varies quite substantially between 3.6 and 19.3. This may be partly due to different source populations and timing of the studies. As our $R_{0}$ was at the lower end of this variation, we think that this was mostly due to a large proportion of once vaccinated (with partial immunity; i.e., asymptomatic or mild infection; hence, lower subsequent infections due to lower virus transmission).

Nowadays, mumps becomes a endemic disease in China and the number of cases tend to be stable each year. There are some connections between the epidemic of mumps and the vacation in China when it is summer and winter. Therefore, the fitting parameters and initial condition of the latter half of year 2013 and the first half of year 2014 may be used to predict the number of clinical cases of the latter half of year 2014 and the first half of year 2015. In fact, the prevalence status of mumps from the last year can be used to predict the epidemic situation of next year.

## 5. Sensitivity Analysis of the Basic Reproduction Number and Endemic Equilibrium

The beginning of a disease transmission is directly related to the basic reproduction number $R_{0}$, while disease prevalence is closely linked with the endemic equilibrium $P^{*}$ [34]. Sensitivity analysis is conducted to identify how closely input parameters that are related to predictor parameter, and determine level of change necessary for an input parameter to acquire the ideal value of a predictor parameter. In order to study the effectiveness of mumps control strategies, we compute the sensitivity indices [34,35,36], which intrinsically measure the relative changes in $R_{0}$ or the state variables at $P^{*}$ with changes of model parameters.

**Definition** **1.** The normalized forward sensitivity index of a variable, u, depending differentiably on a parameter, p, is defined as [34]: $\Delta_{p}^{u}=\frac{p}{u}\times \frac{\partial u}{\partial p}.$

In view of that, it is impossible to control the values of μ, we only compute sensitivity indices of $R_{0}$ and $P^{*}$ with respect to the eight parameters $p_{i}\left(i=1,2,\ldots ,8\right)$: β, $\delta_{1}$, $\delta_{2}$, ε, $\varepsilon_{1}$, λ, α, γ. Here, we consider the parameters from the first half of 2014 (as shown in Table A1). By the way, we consider $\delta_{1}=\delta_{2}=1/12$, according to the Equation (4), it is obvious that γ has no influence on $R_{0}$. Furthermore, the sensitivity indices of $R_{0}$ to the seven parameters, $\Delta_{p_{i}}^{R_{0}}$ are shown in Table 2.

We can observe that β, λ and α ($\delta_{2}$, $\delta_{1}$, ε and $\varepsilon_{1}$) have positive (negative) impacts on $R_{0}$. The most sensitive parameter for $R_{0}$ is β followed by $\delta_{2}$, ε, λ, $\delta_{1}$, α, $\varepsilon_{1}$, (e.g., in order to decrease the value of $R_{0}$ by 1%, it is necessary to decrease the value of β to 1.000000000%).

In addition, one computes the sensitivity indices of the $P^{*}$, which determines the relative impacts of different parameters on disease prevalence. Using the parameters (as shown in Table A1) from the first half of the year 2014 yields

$$P^{*}=\left(0.146893606,0.192406471,0.000461376,0.000054189,0.000238137\right).$$

Then, the sensitivity indices of $P^{*}$ to the eight parameters are calculated in Table 3 with a similar method to Samsuzzoha’s [35], where “+(−)” implies that there are positive (negative) impact on $P^{*}$. Furthermore, some valuable information is obtained: the most sensitive parameter for $S^{*}$ is β followed by $\delta_{2}$, $\delta_{1}$, α, $\varepsilon_{1}$, ε, λ, γ; the most sensitive parameter for $V^{*}$ is ε followed by β, λ, $\delta_{2}$, $\delta_{1}$, α, $\varepsilon_{1}$, γ; the most sensitive parameter for $E^{*}$ is α followed by β, $\delta_{2}$, ε, λ, $\delta_{1}$, $\varepsilon_{1}$, γ; the most sensitive parameter for $I^{*}$ is $\delta_{1}$ followed by γ, β, $\delta_{2}$, ε, λ, α, $\varepsilon_{1}$; the most sensitive parameter for $L^{*}$ is $\delta_{2}$ followed by β, ε, λ, γ, $\delta_{1}$, α, $\varepsilon_{1}$.

## 6. Discussion

According to sensitivity analysis of $R_{0}$ and $P^{*}$ with regard to parameters, several effective measures for mumps’ control and prevention can be put forward and put into practice.

(1) Cut off transmission routes as soon as possible in order to restrain the disease spread among the crowd (reduction β). β is the most sensitive parameter to $R_{0}$. In addition, β is the most sensitive parameter for $S^{*}$, the second sensitive parameter for $V^{*}$, $E^{*}$, $L^{*}$, and the third sensitive parameter for $I^{*}$, respectively. Consequently, parents and teachers should frequently urge students to popularize health knowledge and preserve good personal hygiene habits. Students should wash hands before meals and, after using the toilet, minimize the possibility of getting in touch with other students.

(2) Increasing vaccine coverage (increasing ε=ηφ). ε is the third sensitive to $R_{0}$ and $L^{*}$, and the most sensitive parameter for $V^{*}$. Nevertheless, it is difficult to separately obtain the mumps vaccination rate η and the average vaccination time 1/φ. For most cities and provinces in China, according to the National Immunization Schedule by National Health and Family Planning Commission of the People’s Republic of China (NHFPC) [37], MMR is one of the vaccinations supplied for free by the government, and children just get vaccinated by one dose.

However, as we know, the most common preventative measure against mumps is a vaccination with two doses of the mumps vaccine, applying in many developed countries [22,23]. In the United Kingdom, it is conventional to give children at age 13 months with another dose of vaccine at 3–5 years (preschool). This confers immunity for life. The efficacy of the vaccine depends on the strain of the vaccine, but it is usually around 80% [38]. The American Academy of Pediatrics proposes the routine administration of MMR vaccine at ages 12–15 months and at 4–6 years old [39]. In Canada, provincial governments and the Public Health Agency of Canada have all took part in awareness campaigns to encourage students ranging from the kindergarten to college to get vaccinated. Thus, the Chinese Government, health departments across the country and hospitals should promote teenagers of the right age to continue inoculating with the mumps vaccination.

Of course, if we want to explicate two doses of vaccine accurately, we will need to use pulse model and so on, and this will be our future work.

(3) Recover as soon as possible (decreasing incubation $1/\delta_{1}$ and $1/\delta_{2}$). $\delta_{2}$ is the most sensitive parameter to $L^{*}$, and the second sensitive to $R_{0}$, $S^{*}$. $\delta_{1}$ is the most sensitive parameter for $I^{*}$, the third sensitive parameter for $S^{*}$, respectively. Try to cut down the source of infection. Patients should be treated actively so that they can recover early. Meanwhile, hospitals are supposed to enhance infection control practices to avoid nosocomial cross infection.

(4) Since $\delta_{1}\;=\;\delta_{2}$, γ has no influence on $R_{0}$, but it is a sensitive parameter to $I^{*}$ and $L^{*}$. Thus, we suggest that symptomatic patients should seek medical attention and take necessary precautions to prevent further spread. Actually, 18.02% of persons infected with the mumps virus seek medical attention (seeing Table 1, γ=0.1802), therefore forming the group of severely infectious (*I*). The vast majority, 81.98% of infected mumps patients, do not seek medical attention and form the group (*L*). This group consisted of both mild infections and those completely asymptomatic. From [40], 20–40% of mumps infections may be asymptomatic. That is to say, about 60–80% infections have mild (part of group *L*) or more severe symptoms (mostly in group *I*). From our results, the proportion of patients seeking medical attention (γ) was not high. Therefore, a large proportion of mumps infections are self-treated, thus promoting further spread of the disease.

Owing to the huge and high heterogeneity of the country, in fact, we should consider the spatial stratified heterogeneity of the population and relevant determinants (e.g., climate, temperature, humidity, longitude and latitude, even people’s behavior habits, and so on).

Taking different spatial stratifications, we have reconsidered a new multi-group model Equation (5). The total population *N* is divided into *n* groups, and each subgroup represents different areas. Some parameters in the model Equation (5) are listed below:

- $\beta_{ij}$: rate of disease transmission between susceptible individuals in group *i* and infectious individuals in group *j*,
- $\sigma_{ik}$: transfer rate move from the *k*-th susceptible group to *i*-th susceptible group,
- $\zeta_{il}$: transfer rate move out the *i*-th susceptible group into *l*-th susceptible group,
- $\varphi_{ik}$: transfer rate move from the *k*-th vaccinated group to *i*-th vaccinated group,
- $\upsilon_{il}$: transfer rate move out the *i*-th vaccinated group into *l*-th vaccinated group,
- $\omega_{ik}$: transfer rate move from the *k*-th recovered group to *i*-th recovered group,
- $\phi_{il}$: transfer rate move out the *i*-th recovered group into *l*-th recovered group.

Other parameters of different subgroups are the same as model Equation (1). Due to the incubation period and the duration of the mumps being shorter, we only consider that three categories’ compartments (S,V and *R*) have transfer of each other, and the multi-group model as follows:

(5) $$\begin{array}{c} \left\{\begin{array}{c} \frac{dS_{i}}{dt}=\mu_{i}-\rho_{i}\mu_{i}E-S_{i}\sum_{j=1}^{n}\beta_{ij}\left(I_{i}+L_{i}\right)+\lambda_{i}V_{i}-\left(\varepsilon_{i}+\mu_{i}\right)S_{i}+\sum_{k=1}^{n}\sigma_{ik}S_{k}-\sum_{l=1}^{n}\zeta_{il}S_{i},\\ \frac{dV_{i}}{dt}=\varepsilon_{i}\left(S_{i}+E_{i}\right)-\lambda_{i}V_{i}-\kappa_{i}V_{i}\sum_{j=1}^{n}\beta_{ij}\left(I_{i}+L_{i}\right)-\mu_{i}V_{i}+\sum_{k=1}^{n}\varphi_{ik}V_{k}-\sum_{l=1}^{n}\upsilon_{il}V_{i},\\ \frac{dE_{i}}{dt}=S_{i}\sum_{j=1}^{n}\beta_{ij}\left(I_{i}+L_{i}\right)+\rho_{i}\mu_{i}E_{i}+\kappa_{i}V_{i}\sum_{j=1}^{n}\beta_{ij}\left(I_{i}+L_{i}\right)-\left(\alpha_{i}+\varepsilon_{i}+\mu_{i}\right)E_{i},\\ \frac{dI_{i}}{dt}=\alpha_{i}\gamma_{i}E_{i}-\left(\delta_{1i}+\mu_{i}\right)I_{i},\\ \frac{dL_{i}}{dt}=\alpha_{i}\left(1-\gamma_{i}\right)E_{i}-\left(\delta_{2i}+\mu_{i}\right)L_{i},\\ \frac{dR_{i}}{dt}=\delta_{1i}I_{i}+\delta_{2i}L_{i}-\mu_{i}R_{i}+\sum_{k=1}^{n}\omega_{ik}R_{k}-\sum_{l=1}^{n}\phi_{il}R_{i},\;i=1,2,\cdots ,n. \end{array}\right. \end{array}$$

According to the clinical data from different provinces of China’s CDC [16,17], we can also simulate the parameters of the model. From the analyses of the parameters and the basic reproduction number, we can obtain a lot more meaningful conclusions by studying the data of different spatial and natural environment in different regions. The results of this study will appear in the later papers.

## 7. Conclusions

We have described here an improved autonomous model with a bilinear incidence rate. The model has been developed both in practical and theoretical terms compared to our previous model [23]. The model was concise and we performed extensive sensitivity analysis. The model proved the following theoretical results: (i) by Routh-Herwitz criteria, we proved that, if $R_{0}<1$, the disease-free equilibrium is stable, and $R_{0}>1$, the disease-free equilibrium is unstable; (ii) by LaSalle’s Invariance Principle, we proved the disease-free equilibrium is globally asymptotically stable if $R_{0}\leq 1$; and, (iii) with the help of the Lyapunov function, we proved that, if $R_{0}>1$, for $\rho =\varepsilon_{1}=0$, the unique endemic equilibrium is globally asymptotically stable. Therefore, we would propose the use of this model for further studies.

Using the arithmetic mean of the parameters of model Equation (2), we can calculate $R_{E}=0$, $R_{I}=0.8326$, and $R_{L}=3.7879$. Combining the parameter analysis, one can find that vertical transmission is not the important factor that causes mumps’ sustained outbreaks (ρ was relatively small and almost equal to 0 by our simulation), and young adults with frequent contacts and mild infection were identified as a major driver of mumps epidemics. It can be concluded that a mild infection that carries the virus but doesn’t receive medical attention plays an important part in leading to the epidemic of mumps.

In this work, an SVEILR mumps model incorporating imperfect vaccination, vertical transmission, and mild cases are formulated to describe the dynamics of mumps transmission. As far as we can, we conduct statistical assessments on the sensitivity analysis of $R_{0}$ and the endemic equilibrium $P^{*}$ to parameters, and put forward several corresponding measures to control the spread of mumps. Because of the huge and high heterogeneity of the country, it is important to consider the spatial stratified heterogeneity of the population with relevant determinants (e.g., climate, temperature, humidity, longitude and latitude, even people’s behavior habits, and so on) and choose certain strata of the population. However, in such a huge population it is very difficult to make the strata compatible. We will try to study this problem in the future.

## Figures and Tables

**Table 1:** Parameters values, initial values and the basic reproduction number, where, $\varepsilon ,\varepsilon_{1}$ and S(0) are estimated through calculating the minimum sum of chi-square value (MSCV) in first half of the year (FHY) and fixed in latter half of the year (LHY).

Parameter	Parameter Interval	Median	Arithmetic Mean	Reference
μ	1/(74.83×365)	-	-	[17]
ρ	0	-	-	Fixed
β	0.2933–0.6296	0.4580	0.4421	MSCV
λ	0.0043–0.0254	0.0121	0.0130	MSCV
$\varepsilon =\varepsilon_{1}$	0.0010–0.0057	0.0010	0.0015	MSCV or Fixed
κ	0	-	-	Fixed
α	1/19	-	-	[7,33]
γ	0.0874–0.2933	0.1806	0.1802	MSCV
$\delta_{1}$	1/12	-	-	[9,10,16]
$\delta_{2}$	1/12	-	-	Fixed
S(0)	0.0000–0.5101	0.0027	0.0955	MSCV or Fixed
V(0)	0.4738–0.8629	0.7700	0.7738	MSCV
E(0)	0	-	-	Fixed
I(0)	0	-	-	Fixed
L(0)	0.0161–0.2387	0.1595	0.1307	Calculated
$R_{0}$	2.6529–5.5563	4.7497	4.2806	Calculated

**Table 2:** Sensitivity indices of $R_{0}$, $\Delta_{p_{i}}$, and corresponding % changes in $p_{i}$ to decrease $R_{0}$ by 1%.

Parameter	Sensitivity Indices of $R_{0}$	Corresponding % Changes
β	$\Delta_{\beta }=+1.000000000$	−1.000000000
$\delta_{2}$	$\Delta_{\delta_{2}}=-0.814240098$	+1.228138976
ε	$\Delta_{\varepsilon }=-0.567908157$	+1.760848101
λ	$\Delta_{\lambda }=+0.563084724$	−1.775931680
$\delta_{1}$	$\Delta_{\delta_{1}}=-0.185318088$	+5.396127333
α	$\Delta_{\alpha }=+0.098286622$	−10.17432464
$\varepsilon_{1}$	$\Delta_{\varepsilon_{1}}=-0.097655559$	+10.24007246

**Table 3:** Sensitivity indices of the endemic equilibrium.

Parameter	$S^{*}$	$V^{*}$	$E^{*}$	$I^{*}$	$L^{*}$
β	−1.000150583	−1.002014918	+0.511842285	+0.510066303	+0.509971103
λ	−0.000084504	−0.990736807	+0.287232934	+0.286236298	+0.286182874
ε	+0.000085520	+1.002646670	−0.290685824	−0.289677207	−0.289623141
$\varepsilon_{1}$	+0.097288474	+0.100618754	−0.050701731	−0.050525807	−0.050516377
α	−0.097913587	−0.101242844	−0.948283835	+0.051536721	+0.051527102
γ	$-1.3\times 10^{-17}$	$-1.9\times 10^{-17}$	0	+0.996530216	−0.226764324
$\delta_{1}$	+0.185318305	+0.185663748	−0.094839463	−1.094071232	−0.094492751
$\delta_{2}$	+0.814393053	+0.815911126	−0.416778042	−0.415331912	−1.414815234

**Figure A1:**
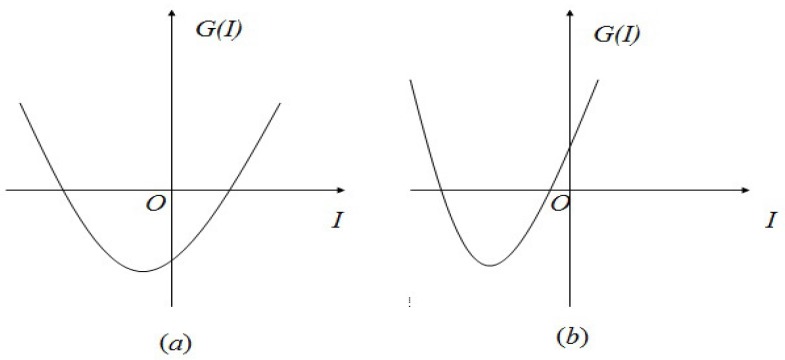
(**a**) shows the graph of G(I) when $R_{0}>1$; (**b**) shows the graph of G(I) when $R_{0}\leq 1$.

## Appendix A.

The endemic equilibrium $P^{*}=\left(S^{*},V^{*},E^{*},I^{*},L^{*}\right)$ of model Equation (2) is determined by equations:

(A1) $$\begin{array}{c} \left\{\begin{array}{c} \mu -\rho \mu E-\beta S(I+L)+\lambda V-(\varepsilon +\mu )S=0,\\ \varepsilon S+\varepsilon_{1}E-\lambda V-\kappa \beta V\left(I+L\right)-\mu V=0,\\ \beta S\left(I+L\right)+\rho \mu E+\kappa \beta V\left(I+L\right)-\left(\alpha +\varepsilon_{1}+\mu \right)E=0,\\ \alpha \gamma E-(\delta_{1}+\mu )I=0,\\ \alpha \left(1-\gamma \right)E-\left(\delta_{2}+\mu \right)L=0. \end{array}\right. \end{array}$$

From the last two equations in Equation (A1), we can obtain

(A2) $$\begin{array}{c} E=\frac{\delta_{1}+\mu }{\alpha \gamma }I,L=\frac{\left(1-\gamma \right)\left(\delta_{1}+\mu \right)}{\gamma (\delta_{2}+\mu )}I, \end{array}$$

when I≠0, substituting Equation (A2) into the third equation in Equation (A1) gives

(A3) $$\begin{array}{c} \beta S+\kappa \beta V=\frac{\left(\varepsilon_{1}+\alpha +\mu -\rho \mu \right)\left(\delta_{1}+\mu \right)\left(\delta_{2}+\mu \right)}{\left(\alpha \gamma \left(\delta_{2}+\mu \right)+\alpha \left(1-\gamma \right)\left(\delta_{1}+\mu \right)\right)}\; \end{array}$$

(A4) $$\begin{array}{c} =\frac{\left(1-R_{E}\right)\left(\beta S_{0}+\kappa \beta V_{0}\right)}{\left(R_{0}-R_{E}\right)}\overset{\Delta }{\;=}d, \end{array}$$

obviously, 0<d<β when $R_{0}>1$.

Substituting Equation (A4) into the first equation in Equation (A1) gives

(A5) $$\begin{array}{c} S=\frac{a_{1}+a_{2}I}{a_{3}+a_{4}I}, \end{array}$$

where

$$a_{1}=\frac{\lambda +\mu }{\kappa \beta }d,\;\;a_{2}=\frac{\left(\delta_{1}+\mu \right)\left(\alpha +\mu -\rho \mu \right)}{\alpha \gamma },$$

$$a_{3}=\varepsilon +\frac{\lambda +\mu }{\kappa },\;\;a_{4}=\beta \left(1+\frac{\left(1-\gamma \right)\left(\delta_{1}+\mu \right)}{\gamma (\delta_{2}+\mu )}\right).$$

Adding together the first, second and third equation in Equation (A1) and obtain

(A6) $$\begin{array}{c} \mu -\mu S-\mu V-(\alpha +\mu )E=0. \end{array}$$

Substituting Equation (A4) into Equation (A6) gives

(A7) $$\begin{array}{c} V=\left(\frac{1}{1-\kappa }\right)\left(1-\frac{d}{\beta }-\frac{\left(\alpha +\mu \right)\left(\delta_{1}+\mu \right)}{\alpha \gamma \mu }I\right), \end{array}$$

(A8) $$\begin{array}{c} S=\left(\frac{1}{1-\kappa }\right)\left(\frac{d}{\beta }-\kappa +\frac{\kappa \left(\alpha +\mu \right)\left(\delta_{1}+\mu \right)}{\alpha \gamma \mu }I\right). \end{array}$$

Substituting Equations (A7) and (A8) into the second equation in Equation (A1) gives

(A9) $$\begin{array}{c} a_{5}I^{2}+a_{6}I+a_{7}\overset{\Delta }{\;=}G\left(I\right)=0, \end{array}$$

where

$$a_{5}=\frac{\kappa \beta \left(\alpha +\mu \right)\left(\delta_{1}+\mu \right)}{\alpha \gamma \mu }\left(1+\frac{\left(1-\gamma \right)\left(\delta_{1}+\mu \right)}{\gamma (\delta_{2}+\mu )}\right)>0,$$

$$a_{6}=\frac{\varepsilon_{1}\left(1-\kappa \right)\left(\delta_{1}+\mu \right)}{\alpha \gamma }-\kappa \beta \left(1+\frac{\left(1-\gamma \right)\left(\delta_{1}+\mu \right)}{\gamma (\delta_{2}+\mu )}\right)\left(1-\frac{d}{\beta }\right)+\frac{\left(\lambda +\mu +\kappa \varepsilon \right)\left(\alpha +\mu \right)\left(\delta_{1}+\mu \right)}{\alpha \gamma \mu },$$

$$\begin{array}{c} a_{7}=\varepsilon \left(\frac{d}{\beta }-\kappa \right)-\left(\lambda +\mu \right)\left(1-\frac{d}{\beta }\right)\;\\ =\frac{1}{\varepsilon +\lambda +\mu }\left(\frac{\left(1-R_{E}\right)\left(S_{0}+\kappa V_{0}\right)}{R_{0}-R_{E}}-S_{0}-\kappa V_{0}\right). \end{array}$$

If $R_{0}>1$, then $a_{7}<0$, so Equation (A9) has a unique positive solution $I=\frac{-a_{6}\;+\;\sqrt{{a_{6}}^{2}-4a_{5}a_{7}}}{2a_{5}}$ (see Figure A1a), and if $R_{0}\leq 1$, then $a_{6}>0$, $a_{7}\geq 0$, Equation (A9) has no positive solution (see Figure A1b). Then, from the Equation (A5), model Equation (2) has a unique positive solution $S^{*}$ when $R_{0}>1$. Similarly, model Equation (2) has a unique positive solution $E^{*},L^{*}$, when $R_{0}>1$. Furthermore, it follows from the second equation in Equation (A1) that $V=\frac{\varepsilon S\;+\;\varepsilon_{1}E}{\lambda \;+\;\mu \;+\;\kappa \beta (I\;+\;L)}$, and we also obtain that model Equation (2) has only one positive solution $V^{*}$ when $R_{0}>1$.

## Appendix B.

**Proof** **of** **Theorem** **1.** The Jacobian matrix at $P_{0}$ is

$$J\left(P_{0}\right)=\left(\begin{array}{ccccc} -(\varepsilon +\mu ) & \lambda & -\rho \mu & -\beta S_{0} & -\beta S_{0}\\ \varepsilon & -(\lambda +\mu ) & \varepsilon_{1} & -\kappa \beta V_{0} & -\kappa \beta V_{0}\\ 0 & 0 & \rho \mu -\varepsilon_{1}-\alpha -\mu & \beta S_{0}+\kappa \beta V_{0} & \beta S_{0}+\kappa \beta V_{0}\\ 0 & 0 & \alpha \gamma & -(\delta_{1}+\mu ) & 0\\ 0 & 0 & \alpha (1-\gamma ) & 0 & -(\delta_{2}+\mu ) \end{array}\right).$$

The characteristic equation is

$$\Phi \left(\xi \right):=\left(\xi^{2}+\left(\lambda +\varepsilon +2\mu \right)\xi +\varepsilon \mu +\lambda \mu +\mu^{2}\right)\left(\xi^{3}+b_{1}\xi^{2}+b_{2}\xi +b_{3}\right)=0,$$

where,

$$b_{1}=\varepsilon_{1}+\alpha +\delta_{1}+\delta_{2}+3\mu -\rho \mu >0,$$

$$b_{2}=\left(\varepsilon_{1}+\alpha +\mu -\rho \mu \right)\left(\delta_{1}+\delta_{2}+2\mu \right)+\left(\delta_{1}+\mu \right)\left(\delta_{2}+\mu \right)-\alpha \beta \left(\kappa V_{0}+S_{0}\right),$$

$$\begin{array}{c} b_{3}=\left(\delta_{1}+\mu \right)\left(\delta_{2}+\mu \right)\left(\varepsilon_{1}+\alpha +\mu -\rho \mu \right)-\alpha \beta \gamma \left(\delta_{2}+\mu \right)\left(\kappa V_{0}+S_{0}\right)\;\\ -\;\alpha \beta \left(1-\gamma \right)\left(\delta_{1}+\mu \right)\left(\kappa V_{0}+S_{0}\right)\;\\ =\left(\delta_{1}+\mu \right)\left(\delta_{2}+\mu \right)\left(\varepsilon_{1}+\alpha +\mu \right)\left(1-R_{0}\right). \end{array}$$

Distinctly, there are two negative solutions of $\xi^{2}+\left(\lambda +\varepsilon +2\mu \right)\xi +\varepsilon \mu +\lambda \mu +\mu^{2}$, while $R_{0}<1$, $b_{3}>0$. In addition,

$$\begin{array}{c} b_{1}b_{2}-b_{3}=\left(\delta_{1}+\mu \right)\left(\delta_{2}+\mu \right)\left(\delta_{1}+\delta_{2}+2\mu \right)\;\\ +\;\left(\varepsilon_{1}+\alpha +\delta_{1}+\delta_{2}+3\mu -\rho \mu \right)\left(\varepsilon_{1}+\alpha +\mu -\rho \mu \right)\left(\delta_{1}+\delta_{2}+2\mu \right)\;\\ +\;\alpha \beta \gamma \left(\varepsilon_{1}+\alpha +\delta_{1}+2\mu -\rho \mu \right)\left(\kappa V_{0}+S_{0}\right)\;\\ +\;\alpha \beta \left(1-\gamma \right)\left(\varepsilon_{1}+\alpha +\delta_{2}+2\mu -\rho \mu \right)\left(\kappa V_{0}+S_{0}\right)>0. \end{array}$$

Therefore, by Routh-Herwitz criteria, all roots of Φ(ξ) have negative real parts, so $P_{0}$ is stable. If $R_{0}>1$, then $b_{3}<0$, and furthermore $P_{0}$ is unstable. ☐

**Proof** **of** **Theorem** **2.** Consider the following Lyapunov function:

$$H=c_{1}E+c_{2}I+c_{3}L,$$

where

$$c_{1}=\alpha \gamma \left(\delta_{2}+\mu \right)+\alpha \left(1-\gamma \right)\left(\delta_{1}+\mu \right),c_{2}=\left(\delta_{2}+\mu \right)\left(\varepsilon_{1}+\alpha +\mu -\rho \mu \right)$$

$$c_{3}=\left(\delta_{1}+\mu \right)\left(\varepsilon_{1}+\alpha +\mu -\rho \mu \right).$$

The derivative of *H* along the solutions of model Equation (2), together with $S\leq S_{0}$ and $V\leq V_{0}$, yields

$$\dot{H}=c_{1}\dot{E}+c_{2}\dot{I}+c_{3}\dot{L}=$$

$$=c_{1}\beta SI+c_{1}\beta SL-c_{1}\left(\varepsilon_{1}+\alpha +\mu -\rho \mu \right)E+c_{1}\kappa \beta VI+c_{1}\kappa \beta VL$$

$$+\;c_{2}\alpha \gamma E-c_{2}\left(\delta_{1}+\mu \right)I+c_{3}\alpha \left(1-\gamma \right)E-c_{3}\left(\delta_{2}+\mu \right)L$$

$$\leq c_{1}\left(\kappa \beta V_{0}+\beta S_{0}\right)I-c_{2}\left(\delta_{1}+\mu \right)I+c_{1}\left(\kappa \beta V_{0}+\beta S_{0}\right)L-c_{3}\left(\delta_{2}+\mu \right)L$$

$$+\;c_{2}\alpha \gamma E-c_{1}\left(\varepsilon_{1}+\alpha +\mu -\rho \mu \right)E+c_{3}\alpha \left(1-\gamma \right)E$$

$$=\left(\varepsilon_{1}+\alpha +\mu \right)\left(\delta_{1}+\mu \right)\left(\delta_{2}+\mu \right)\left(R_{0}-1\right)\left(I+L\right).$$

Since all of the parameters are positive, it follows that $\dot{H}\leq 0$ for $R_{0}\leq 1$. There exists a singleton $P_{0}$, as the maximal compact invariant set in the set $\left\{\left(S,V,E,I,L\right)\in \Omega :\dot{H}=0\right\}$. Therefore, by LaSalle’s Invariance Principle [41], every solution of model Equation (2), with initial conditions in Ω approaches $P_{0}$ as t→∞, whenever $R_{0}\leq 1$. This completes the proof. ☐

**Proof** **of** **Theorem** **3.** Let

$$x=\frac{S}{S^{*}},y=\frac{V}{V^{*}},z=\frac{E}{E^{*}},u=\frac{I}{I^{*}},v=\frac{L}{L^{*}}.$$

When $\rho =\varepsilon_{1}=0$, the model Equation (2) is transformed into the following form:

(A10) $$\begin{array}{c} \left\{\begin{array}{c} \frac{dx}{dt}=x\left(\frac{\mu }{S^{*}}\left(\frac{1}{x}-1\right)+\frac{\lambda V^{*}}{S^{*}}\left(\frac{y}{x}-1\right)-\beta I^{*}\left(u-1\right)-\beta L^{*}\left(v-1\right)\right),\\ \frac{dy}{dt}=y\left(\frac{\varepsilon S^{*}}{V^{*}}\left(\frac{x}{y}-1\right)-\kappa \beta I^{*}\left(u-1\right)-\kappa \beta L^{*}\left(v-1\right)\right),\\ \frac{dz}{dt}=z\left(\frac{\beta S^{*}I^{*}}{E^{*}}\left(\frac{xu}{z}-1\right)+\frac{\beta S^{*}L^{*}}{E^{*}}\left(\frac{xv}{z}-1\right)+\frac{\kappa \beta V^{*}I^{*}}{E^{*}}\left(\frac{yu}{z}-1\right)+\frac{\kappa \beta V^{*}L^{*}}{E^{*}}\left(\frac{yv}{z}-1\right)\right),\\ \frac{du}{dt}=u\frac{\alpha \gamma E^{*}}{I^{*}}\left(\frac{z}{u}-1\right),\\ \frac{dv}{dt}=v\frac{\alpha \left(1-\gamma \right)E^{*}}{L^{*}}\left(\frac{z}{v}-1\right). \end{array}\right. \end{array}$$

It is easy to find that model Equation (A10) has a unique endemic equilibrium ${P_{1}}^{*}\left(1,1,1,1,1\right)$, and the global stability of ${P_{1}}^{*}$ is the same as that of $P^{*}$. Thus, we will investigate the global stability ${P_{1}}^{*}$ instead of $P^{*}$. Defining the Volterra-type Lyapunov function

$$H\left(x,y,z,u,v\right)=S^{*}\left(x-1-\ln x\right)+V^{*}\left(y-1-\ln y\right)+E^{*}\left(z-1-\ln z\right)$$

$$+\frac{\beta I^{*}\left(S^{*}+\kappa V^{*}\right)}{\alpha \gamma E^{*}}\left(u-1-\ln u\right)+\frac{\beta L^{*}\left(S^{*}+\kappa V^{*}\right)}{\alpha \left(1-\gamma \right)E^{*}}\left(v-1-\ln v\right).$$

For the equilibrium state $P^{*}$, we have the following equations:

(A11) $$\begin{array}{c} \left\{\begin{array}{c} \mu +\lambda V^{*}=\beta S^{*}\left(I^{*}+L^{*}\right)+\left(\varepsilon +\mu \right)S^{*},\\ \varepsilon S^{*}=\left(\lambda +\mu \right)V^{*}+\kappa \beta V^{*}\left(I^{*}+L^{*}\right),\\ \beta S^{*}\left(I^{*}+L^{*}\right)+\rho \mu E^{*}+\kappa \beta V^{*}\left(I^{*}+L^{*}\right)=\left(\alpha +\mu \right)E^{*},\\ \alpha \gamma E^{*}=\left(\delta_{1}+\mu \right)I^{*},\\ \alpha \left(1-\gamma \right)E^{*}=\left(\delta_{2}+\mu \right)L^{*}. \end{array}\right. \end{array}$$

Then, differentiating *H* with respect to *t* along solution curves of model Equation (A10) and considering Equation (A11) gives

$$\begin{array}{c} \frac{dH}{dt}=S^{*}\left(x-1\right)\frac{\dot{x}}{x}+V^{*}\left(y-1\right)\frac{\dot{y}}{y}+E^{*}\left(z-1\right)\frac{\dot{z}}{z}+\frac{\beta I^{*}\left(S^{*}+\kappa V^{*}\right)}{\alpha \gamma E^{*}}\left(u-1\right)\frac{\dot{u}}{u}+\frac{\beta L^{*}\left(S^{*}+\kappa V^{*}\right)}{\alpha \left(1-\gamma \right)E^{*}}\left(v-1\right)\frac{\dot{v}}{v}\\ =\left(x-1\right)\left(\mu \left(\frac{1}{x}-1\right)+\lambda V^{*}\left(\frac{y}{x}-1\right)-\beta S^{*}I^{*}\left(u-1\right)-\beta S^{*}L^{*}\left(v-1\right)\right)\\ +\;\left(y-1\right)\left(\varepsilon S^{*}\left(\frac{x}{y}-1\right)-\kappa \beta V^{*}I^{*}\left(u-1\right)-\kappa \beta V^{*}L^{*}\left(v-1\right)\right)\\ +\;\left(z-1\right)\left(\beta S^{*}I^{*}\left(\frac{xu}{z}-1\right)+\beta S^{*}L^{*}\left(\frac{xv}{z}-1\right)+\kappa \beta V^{*}I^{*}\left(\frac{yu}{z}-1\right)+\kappa \beta V^{*}L^{*}\left(\frac{yv}{z}-1\right)\right)\\ +\;\beta I^{*}\left(S^{*}+\kappa V^{*}\right)\left(u-1\right)\left(\frac{z}{u}-1\right)+\beta L^{*}\left(S^{*}+\kappa V^{*}\right)\left(v-1\right)\left(\frac{z}{v}-1\right)\\ =2\mu +\lambda V^{*}+\varepsilon S^{*}+\beta I^{*}\left(S^{*}+\kappa V^{*}\right)+\beta L^{*}\left(S^{*}+\kappa V^{*}\right)\\ -\;\mu S^{*}x-\mu V^{*}y-\mu \frac{1}{x}-\lambda V^{*}\frac{y}{x}-\varepsilon S^{*}\frac{x}{y}-\beta S^{*}I^{*}\frac{xu}{z}-\beta S^{*}L^{*}\frac{xv}{z}\\ -\;\kappa \beta V^{*}I^{*}\frac{yu}{z}-\kappa \beta V^{*}L^{*}\frac{yv}{z}-\left(\beta S^{*}I^{*}+\kappa \beta V^{*}I^{*}\right)\frac{z}{u}-\left(\beta S^{*}L^{*}+\kappa \beta V^{*}L^{*}\right)\frac{z}{v}. \end{array}$$

After some algebraic manipulations, we have

$$\begin{array}{c} \frac{dH}{dt}=\mu S^{*}\left(2-x-\frac{1}{x}\right)+\lambda V^{*}\left(2-\frac{y}{x}-\frac{x}{y}\right)+\mu V^{*}\left(3-\frac{1}{x}-y-\frac{x}{y}\right)+\beta S^{*}I^{*}\left(3-\frac{1}{x}-\frac{xu}{z}-\frac{z}{u}\right)\;\\ +\;\beta S^{*}L^{*}\left(3-\frac{1}{x}-\frac{xv}{z}-\frac{z}{v}\right)+\kappa \beta V^{*}I^{*}\left(4-\frac{1}{x}-\frac{x}{y}-\frac{yu}{z}-\frac{z}{u}\right)+\kappa \beta V^{*}L^{*}\left(4-\frac{1}{x}-\frac{x}{y}-\frac{yv}{z}-\frac{z}{v}\right).\;\\ \leq 0 \end{array}$$

The equality $\frac{dH}{dt}=0$ holds for x=y=1,z=u=v , which corresponds to the set $\bar{\Omega }=\left\{\left(S,V,E,I,L\right):S=S^{*},V=V^{*},\frac{E}{E^{*}}=\frac{I}{I^{*}}=\frac{L}{L^{*}}\right\}\subset \Omega$. This means that $P^{*}$ is the maximum invariant set of model Equation (2) in $\bar{\Omega }$, and then the endemic equilibrium $P^{*}$ is global asymptotic stability in Ω. ☐

For the full model, we guess that the endemic equilibrium $P^{*}$ is also globally stable as $R_{0}>1$. To prove this result, we may need new Lyapunov function or technique. This will be our future work.

## Appendix C.

### Appendix C.1. Parameter Estimation

We estimate parameters of system (1) by calculating the minimum sum of chi-square value (MSCV) [30]:

$$\begin{array}{c} J\left(\theta \right)=\sum_{i=1}^{m}\frac{{\left(I\left(t_{i}\right)-\hat{I}\left(t_{i}\right)\right)}^{2}}{\hat{I}\left(t_{i}\right)},m=6\;\mathrm{or}\;8, \end{array}$$

with the MATLAB (The Mathworks, Inc., Natick, MA, USA) tool fminsearch, where $I(t_{i})$ denotes the actual data of the *i* month, and $\hat{I}\left(t_{i}\right)$ denotes the simulative results of severe infections requiring medical attention of the *i* month. All optimal parameter values are obtained only when the results of fminsearch are convergent. Those values are shown in Table A1 and Table A2. Here, we also calculate the basic reproduction number.

**Table A1:** Estimation of parameters and the basic reproduction number of the first half of the year (FHY, e.g., 09(2–9) stands for February to September of 2009, and MSCV stands for the minimum sum of chi-square value.) from 2009 to 2014.

Parameter Interval and $R_{0}$	Source	09(2–9)	10(2–9)	11(2–9)	12(2–9)	13(2–9)	14(2–9)
ρ	Fixed	0	0	0	0	0	0
β∈ [0.0001,50]	MSCV	0.4630	0.5368	0.4580	0.3848	0.2933	0.6296
λ∈ [0.0001,0.1]	MSCV	0.0095	0.0073	0.0120	0.0150	0.0069	0.0043
$\varepsilon_{1}=\varepsilon_{2}\in$ [0.001,1]	MSCV	0.0010	0.0010	0.0014	0.0010	0.0019	0.0057
κ	Fixed	0	0	0	0	0	0
α∈ [1/15,1/24]	Fixed	1/19	1/19	1/19	1/19	1/19	1/19
γ∈ [0.01,1]	MSCV	0.1967	0.1750	0.2664	0.2933	0.2172	0.1854
$\delta_{1}=\delta_{2}\in$ [1/14,1/10]	Fixed	1/12	1/12	1/12	1/12	1/12	1/12
S(0)	MSCV	0.1680	0.1037	0.0000	0.0027	0.5101	0.2150
V(0)	MSCV	0.7644	0.8548	0.8406	0.8356	0.4738	0.7646
E(0)	Fixed	0	0	0	0	0	0
I(0)	Fixed	0	0	0	0	0	0
L(0)	Calculated	0.0677	0.0415	0.1595	0.1617	0.0161	0.0203
R(0)	Fixed	0	0	0	0	0	0
$R_{0}$	Calculated	4.9302	5.5563	4.8079	4.2449	2.6529	2.9525

**Table A2:** Estimation of parameters and the basic reproduction number of the latter half of the year (LHY, e.g., 09(9)–10(2) stands for September of 2009 to February of 2010, and MSCV stands for the minimum sum of chi-square value.) from 2009 to 2014.

Parameter Interval and $R_{0}$	Source	09(9)–10(2)	10(9)–11(2)	11(9)–12(2)	12(9)–13(2)	13(9)–14(2)
ρ	Fixed	0	0	0	0	0
β∈ [0.0001,50]	MSCV	0.4400	0.3062	0.4256	0.3758	0.5499
λ∈ [0.0001,0.1]	MSCV	0.0121	0.0254	0.0185	0.0169	0.0149
$\varepsilon_{1}=\varepsilon_{2}\in$ [0.001,1]	Fixed	0.0010	0.0010	0.0010	0.0010	0.0010
κ	Fixed	0	0	0	0	0
α∈ [1/15,1/24]	Fixed	1/19	1/19	1/19	1/19	1/19
γ∈ [0.01,1]	MSCV	0.0928	0.1162	0.1708	0.1806	0.0874
$\delta_{1}=\delta_{2}\in$ [1/14,1/10]	Fixed	1/12	1/12	1/12	1/12	1/12
S(0)	Fixed	0.0469	0.0025	0.0019	0.0000	0.0000
V(0)	MSCV	0.7462	0.8629	0.8371	0.7613	0.7700
E(0)	Fixed	0	0	0	0	0
I(0)	Fixed	0	0	0	0	0
L(0)	Calculated	0.2068	0.1345	0.1611	0.2387	0.2298
R(0)	Fixed	0	0	0	0	0
$R_{0}$	Calculated	4.7807	3.4661	4.7497	4.1738	4.7720

**Table A3:** Chi-square values and degrees of freedom for the first half of the year (FHY, e.g., 09(2–9) stands for February to September of 2009, and υ denotes degrees of freedom and AV denotes the accepting value at a 5% significant level with degrees of freedom 1).

	09(2–9)	10(2–9)	11(2–9)	12(2–9)	13(2–9)	14(2–9)
chi-square value	0.0112	0.0031	0.0065	0.0084	0.0048	0.0031
υ	1	1	1	1	1	1
AV	3.841	3.841	3.841	3.841	3.841	3.841

**Table A4:** Chi-square values and degrees of freedom for the latter half of the year (LHY, e.g., 09(9)–10(2) stands for September of 2009 to February of 2010, and υ denotes degrees of freedom and AV denotes the accepting value at a 5% significant level with degrees of freedom 1).

	09(9)–10(2)	10(9)–11(2)	11(9)–12(2)	12(9)–13(2)	13(9)–14(2)
chi-square value	0.0009	0.0010	0.0010	0.0016	$1.0879\times 10^{-6}$
υ	1	1	1	1	1
AV	3.841	3.841	3.841	3.841	3.841

### Appendix C.2. Chi-Square Test of Goodness of Fit

In order to test how well our model reflects the data actually, we consider the following hypotheses:

**Hypothesis** **A1** (*H*_*A*1_) Null hypothesis: the estimated parameters are equal to actual values.

**Hypothesis** **A1** (*H*_*A*2_) Alternative hypothesis: the estimated parameters are not equal to actual values.

The chi-square values and degrees of freedom for each year are shown in Table A3 and Table A4. It is obvious that all chi-square values are much less than 3.841. Therefore, we can not reject the null hypothesis at the 5% significant level by Pearson’s criterion of a chi-square test [42].
